# Marker-Assisted Pyramiding of Blast-Resistance Genes in a *japonica* Elite Rice Cultivar through Forward and Background Selection

**DOI:** 10.3390/plants12040757

**Published:** 2023-02-08

**Authors:** Elisa Zampieri, Andrea Volante, Caterina Marè, Gabriele Orasen, Francesca Desiderio, Chiara Biselli, Marco Canella, Lorena Carmagnola, Joëlle Milazzo, Henri Adreit, Didier Tharreau, Nicolas Poncelet, Patrizia Vaccino, Giampiero Valè

**Affiliations:** 1 Council for Agricultural Research and Economics—Research Centre for Cereal and Industrial Crops, s.s. 11 to Torino, km 2.5, 13100 Vercelli, VC, Italy; 2 Institute for Sustainable Plant Protection, National Research Council, Strada Delle Cacce 73, 10135 Turin, TO, Italy; 3 Council for Agricultural Research and Economics—Research Centre for Vegetable and Ornamental Crops, Corso Inglesi 508, 18038 Sanremo, IM, Italy; 4 Council for Agricultural Research and Economics—Research Centre for Genomics and Bioinformatics, Via S. Protaso 302, 29017 Fiorenzuola d’Arda, PC, Italy; 5 Bertone Sementi S.P.A., Strada Cacciolo, 15030 Terruggia, AL, Italy; 6 Council for Agricultural Research and Economics—Viticulture and Oenology, Viale Santa Margherita 80, 52100 Arezzo, AR, Italy; 7 CIRAD, UMR PHIM TA A 120/K, Campus de Baillarguet, 34, CEDEX 5, 34398 Montpellier, France; 8 Plant Health Institute of Montpellier (PHIM), University of Montpellier, CIRAD, INRAE, IRD, Montpellier SupAgro, 34, 34398 Montpellier, France; 9 Dipartimento per lo Sviluppo Sostenibile e la Transizione Ecologica, Università del Piemonte Orientale, Piazza San Eusebio 5, 13100 Vercelli, VC, Italy

**Keywords:** *Pyricularia oryzae*, *Pi* genes, pyramiding, backcross, rice, molecular markers, breeding, KASP marker

## Abstract

Rice blast, caused by *Pyricularia oryzae*, is one of the main rice diseases worldwide. The pyramiding of blast-resistance (*Pi*) genes, coupled to Marker-Assisted BackCrossing (MABC), provides broad-spectrum and potentially durable resistance while limiting the donor genome in the background of an elite cultivar. In this work, MABC coupled to foreground and background selections based on KASP marker assays has been applied to introgress four *Pi* genes (*Piz, Pib, Pita*, and *Pik*) in a renowned *japonica* Italian rice variety, highly susceptible to blast. Molecular analyses on the backcross (BC) lines highlighted the presence of an additional blast-resistance gene, the *Pita*-linked *Pita2*/*Ptr* gene, therefore increasing the number of blast-resistance introgressed genes to five. The recurrent genome was recovered up to 95.65%. Several lines carrying four (including *Pita2*) *Pi* genes with high recovery percentage levels were also obtained. Phenotypic evaluations confirmed the effectiveness of the pyramided lines against multivirulent strains, which also had broad patterns of resistance in comparison to those expected based on the pyramided *Pi* genes. The developed blast-resistant *japonica* lines represent useful donors of multiple blast-resistance genes for future rice-breeding programs related to the *japonica* group.

## 1. Introduction

Rice blast, caused by the ascomycete *Pyricularia oryzae* (synonym *Magnaporthe oryzae,* [1,2]), is one of the most damaging fungal diseases of rice at the World scale [3]. It causes lesions to all aerial parts of the plant, mainly leaves and panicles, resulting in yield losses, sometimes up to 100% under favorable environmental conditions [4] and equivalent to the food of 60 million people annually [5]. Most of the current European varieties show intermediate or low resistance to blast in field conditions; in particular, some traditional renowned Italian varieties currently cultivated, such as Carnaroli and Vialone Nano, are highly susceptible [6,7,8].

Blast disease control by chemicals increases production costs and impacts the environment, favoring the emergence and selection of new fungal strains resistant to fungicides. Furthermore, some molecules used in chemical control pose health issues that are subject to scrutiny by competent authorities. For example, the residues of tricyclazole were limited to 0.01 mg/kg of grains by EU ruling in 2017, resulting in the banning of its use. Recently, control agencies have also imposed a progressive reduction in chemical employment on the basis of Directive 2009/128/CE, encouraging researchers to develop alternative solutions.

The generation of resistant rice varieties, harboring blast-resistance (*R*) genes (*Pi* genes [7,9,10,11]), has gained a central role in breeding programs worldwide. To date, more than 146 blast *Pi* genes and 500 quantitative trait loci (QTLs) have been identified, and about 36 of them have been cloned [9,12,13,14], providing the opportunity to introgress two or more resistant loci into a unique line by means of the gene-pyramiding strategy [7,15,16,17]. This approach allows a broad-spectrum resistance to be obtained and the risk of resistance breakdown by the pathogen to be reduced [18,19,20].

An ideal pyramiding product should contain as little donor genome as possible in the genetic background of an elite recurrent variety. Recurrent backcrossing was a traditional breeding method employed to transfer alleles from a donor to an elite cultivar (cv), with an expected recurrent parent (RP) genome of 99.2% after at least six backcrosses [21,22]. An alternative approach to introgress genes of interest into an elite variety is Marker-Assisted BackCrossing (MABC), based on the combination of backcrosses and genetic screenings with molecular markers. In this way, it is possible to track the specific resistance *loci* in segregating populations avoiding phenotypic screening [23] and to recover the RP genome in less time (by three backcrosses) in comparison to conventional breeding [22,24,25,26]. In brief, two steps, i.e., foreground selection and background selection, are applied. Foreground selection consists of the analysis of specific markers associated (closely located or positioned inside) with the target genes, while background selection is based on dispersed markers throughout the genome for the recovery of the RP genetic background [27]. Several molecular markers associated with *Pi* genes have been developed and tested on different donors and potential receivers represented by *japonica* varieties [7,20]. Moreover, the level of resistance against the Northern Italy field-blast population conferred by different *Pi* genes has been evaluated, allowing the identification of *Pi* genes (including *Pita, Pik*, *Piz,* and *Pib*) that are effective against the field strains of the pathogen [7]. Unlike the other-mentioned *Pi* genes, the function of the *Pik* locus relies on the presence of two genes, *Pik-1* and *Pik-2*, which are not homologous but are adjacent in the genome, separated by only about 2,5 kb and both required to confer *Pik* resistance [28]. PIK-1 contains an integrated heavy-metal-associated (HMA) domain that is responsible for the direct binding of the AVR-PIK effector [29,30]. Many non-synonymous nucleotide polymorphisms have been observed at *Pik-1* among rice cvs [29,30], and some of them have occurred at the HMA domain-encoding sequence, most likely contributing to recognition specificity [31]. Conversely, *Pik-2* shows a low level of DNA polymorphism among different genotypes [31].

The present work describes the pyramiding of four *Pi* genes (*Pib, Pik*, *Pita,* and *Piz*), previously demonstrated as effective against the field-blast population in Italy, in the genetic background of the highly susceptible renowned Italian cv Vialone Nano, by a MABC-based program. The four target *Pi* genes are located on rice chromosomes 2 (*Pib*) [13,32], 6 (*Piz*) [33], 11 (*Pik*) [28,34], and 12 (*Pita*) [34,35]. The donor line for the four *Pi* genes, named SJKK, was obtained from a pyramiding program assisted by molecular markers using Saber, Jefferson, Katy, and Kusabue as donors for *Pib*, *Piz*, *Pita*, and *Pik*, respectively [17]. The obtained backcross (BC) lines showed up to 95.2% of the RP genome, and phenotypic evaluations of blast resistance demonstrated that the presence of multiple *Pi* genes conferred valuable resistance levels against four multivirulent *P. oryzae* strains.

## 2. Results

### 2.1. Development of Molecular Markers

*Pi* genes were introgressed in the cv Vialone Nano by crossing with the donor line SJKK. Diagnostic markers for *Piz* and *Pib* were obtained from previous publications (Table 1 [7,33,36]) and were used to amplify the genomic DNA from the original donors (Saber and Jefferson, respectively, for *Pib* and *Piz*) and the parents SJKK and Vialone Nano.

Sequencing of the *Pib* marker amplicons with primers Pib5f/r allowed the identification of a [C/T] Single Nucleotide Polymorphism (SNP) between the *Pib* donor SJKK and Vialone Nano (Table 2; Supplementary Figure S1); Saber carried the same allele as SJKK at this locus. For the *Piz* marker amplicon, sequencing with Z56592f/r showed a [G/A] SNP between the *Piz* donor SJKK and Vialone Nano (Table 2; Supplementary Figure S1), with Jefferson showing the same allele as SJKK at this locus.

**Figure 1:**
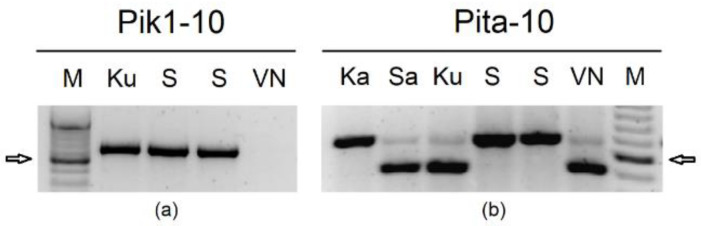
Molecular markers developed in this work for *Pik1* (**a**) and *Pita* (**b**). M = molecular weight marker; Ku = Kusabue (*Pik* original donor); S = SJKK (donor parent); Sa = Saber; Ka = Katy (*Pita* original donor); VN = Vialone Nano (recurrent parent). Arrows indicate 500 bp size.

Diagnostic markers for *Pik1* were developed in this work. The sequencing of the *Pik* locus LOC_Os11g46200 (Os11g0688832) in the original donor Kusabue, SJKK, and Vialone nano allowed the identification of a dominant pres/abs marker, defined by primers Pik1-10f/r, in the donor and receiving genotypes (Figure 1a).

Since no amplicons were obtained for the Vialone Nano genome with the whole set of Pik1 primer pairs (Supplementary Table S1), it could be hypothesized either that this gene is absent in this susceptible genotype or that its sequence is largely divergent. Pik1-10 was therefore useful during the BC generations, but did not discriminate homo- or heterozygous genotypes in the selfing generations. Since no sequences could be obtained from Vialone Nano to generate co-dominant markers, *Pik2* (GenBank accession number HM048900, LOC_Os11g46210, Os11g0689100; [34]) was sequenced in Vialone Nano, Kusabue, and SJKK; and a diagnostic marker, based on a [G/A] SNP between SJKK (and the original donor Kusabue) and Vialone Nano and corresponding to the primer combination Pik2-2AEf/r, was developed (Table 2; Supplementary Figure S1). The availability of two markers for *Pik*, one dominant for Pik1 (Pik1-10f/r) and one co-dominant for Pik2 (Pik2-2AEf/r), allowed selection for both genes in two different steps of the process: Pik1-10 was used during the backcrosses, while Pik2-2AE was used for selecting the homozygous F_2_ plants (as explained below). This procedure reinforced the selection power, allowing the identification of F_2_ lines carrying resistant alleles for both genes at the *Pik* locus. In Kusabue the two genes are tightly physically linked on chromosome 11 (separated by 2543 bp; [34]), making the possibility of recombination among the two genes during the backcrossing process extremely unlikely.

The *Pita* locus was sequenced in both parents and in the original donor Katy; primers were designed from bp 10,606,359 to bp 10,612,068 of LOC_Os12g18360 (Os12g0281300) [35]. The amplification with primers Pita-10f/r allowed the identification of a [T/A] SNP between SJKK and Vialone Nano (Table 2; Supplementary Figure S1); the same allele was identified in the original donor Katy. This SNP was exploited to obtain a HincII Cleaved Amplified Polymorphic Sequence (CAPS) marker (Figure 1b).

**Figure 2:**
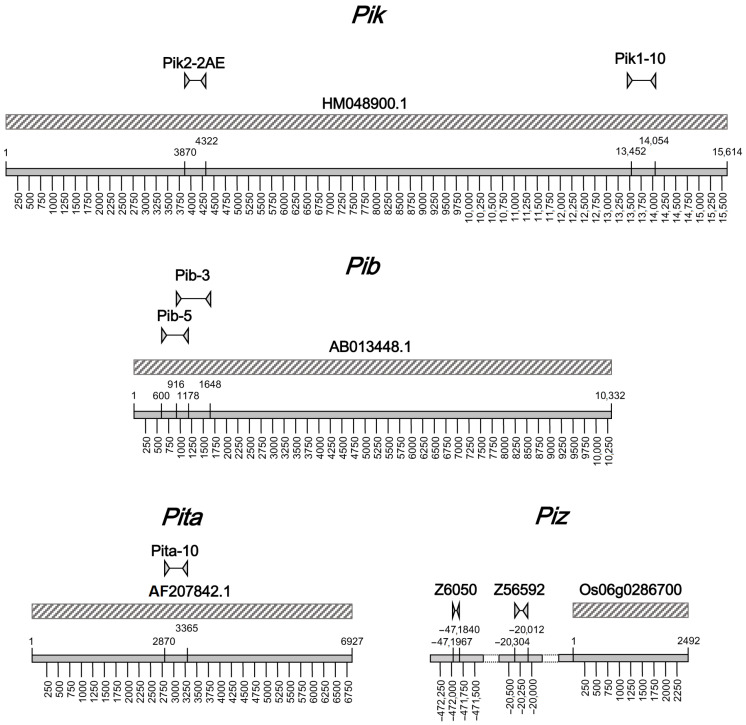
Position of the *Pi* markers listed in Table 1 and Table 2 in the *Pik*, *Pib*, *Pita*, and *Piz* homolog genes on Nipponbare reference genome IRGSP-1.0 (GenBank accessions HM048900.1 (LOC_Os11g46210, Os11g0689100), AB013448.1 (LOC_Os02g57305, Os02g0818450), AF207842.1 (LOC_Os12g18360, Os12g0281300), and RAP-DB accession Os06g0286700 (LOC_Os06g17900), respectively).

The position of the developed markers on the *Pik*, *Pib*, *Pita*, and *Piz* homologous genes in the Nipponbare reference genome is showed in Figure 2.

### 2.2. MABC

**Figure 3:**
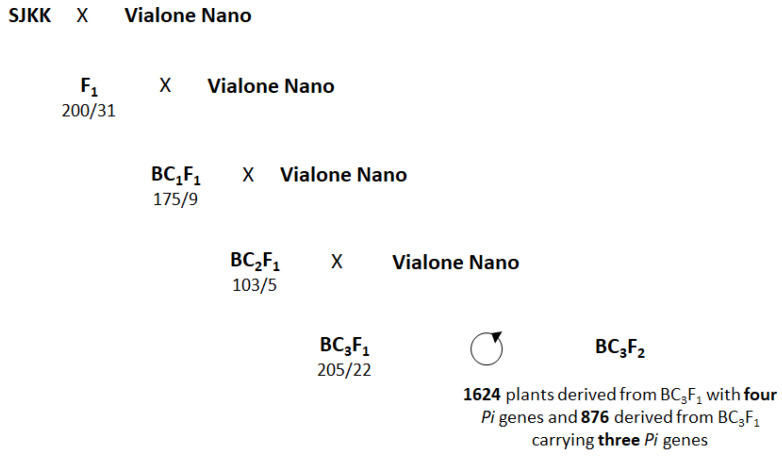
MABC scheme for the introgression of the four *Pi* genes (*Pib*, *Piz*, *Pik*, and *Pita*) where SJKK acted as donor parent (DP) and Vialone Nano as recurrent parent (RP). Numbers indicate the tested plants with respect to the number of plants having the desired marker alleles.

The MABC program started with a cross between the donor parent (DP) SJKK and Vialone Nano (RP). F_1_ plants were tested for being produced from a cross rather than from selfing by using the *Pib* markers listed in Table 1. Thirty-one plants were identified as true F_1_ (Figure 3) and were backcrossed to the RP, obtaining 175 BC_1_F_1_ lines that were tested with the markers for the four *Pi* genes (Pib3; Z6050; Pik1-10; Pita-10).

Nine lines harboring the four *Pi* genes in heterozygosity were identified out of 175, following approximately the expected percentage of 6.25%. These plants were backcrossed again with the RP, and 103 BC_2_F_1_ lines were obtained. Marker screening revealed that five of them were heterozygous at the four *Pi* loci, again approaching the expected percentage of 6.25%. After the third backcross, 205 BC_3_F_1_ lines were analyzed to recover heterozygous lines: seven of these harbored the four *Pi* genes, while fifteen carried three genes in two different combinations (*Pita + Pib + Piz* and *Pita + Pib + Pik*). All 22 plants were self-pollinated to obtain BC_3_F_2_ seeds, resulting in the production of 1624 plants from the BC_3_F_1_ lines carrying the four *Pi* genes and 876 BC_3_F_2_ plants from the BC_3_F_1_ lines with three *Pi* genes, accounting for a total of 2500 BC_3_F_2_ individuals. The BC_3_F_2_ plants were subsequently analyzed in foreground selection to identify homozygous plants in the four or three *Pi* loci.

### 2.3. Foreground Selection

The 2500 BC_3_F_2_ plants were analyzed by Kompetitive Allele Specific (KASP) marker assays together with the parents SJKK and Vialone Nano. Co-dominant KASP markers based on SNPs between the DP and the RP (Table 2) were utilized for the screening. The assays were pursued through the utilization of “on-the-gene” co-dominant markers for *Pik* (Pik2-2AE), *Pib* (Pib5), and *Pita* (Pita-10), and a co-dominant marker (Z56592) for *Piz* that was localized at about 20 Kbp from the target gene on a Nipponbare reference genome (Figure 2).

Of the original 2500 BC_3_F_2_ lines, 2364 produced seeds. Application of the results of the foreground assay to these plants allowed the identification of homozygous plants for resistant alleles of the *Pi* genes, i.e., 8 plants with four *Pi* genes, 82 with three genes, 371 with two genes, and 839 with one gene (Table 3; Supplementary Table S2).

The percentage of lines carrying the four *Pi* genes at the homozygous state (0.34%) was in agreement with the expected frequency of 1/256 (0.39%) for the segregation of four genes in an F_2_ population. For the combinations of three genes, the expected homozygosity ratio of 1/64 (1.56%) was approximately observed for *Piz + Pib + Pita* (1.1%) and *Pib + Pik + Pita* (1.7%), while for *Piz + Pik + Pita* (0.42%) and *Piz + Pib + Pik* (0.21%) a lower-than-expected homozygosity ratio was observed. For the combinations of two genes, the expected homozygosity ratio of 1/16 (6.25%) was approximately observed only for *Pib + Pita* (6.2%), while for all the other combinations a lower than expected value of homozygous lines occurred: 0.8% for *Piz + Pik*; 2.16% for *Piz + Pita*; 1.9% for *Pib + Pik*; 1.9% for *Piz + Pita*; 2.7% for *Pik + Pita*. All the lines with only one *Pi* gene showed a segregation that yielded a lower number of homozygous lines than expected (25%).

### 2.4. Background Selection

Ninety-six KASP markers, among those commercially available at LGC Genomics, were selected for the 12 rice chromosomes. On average, 7 markers were located on chromosomes not carrying *Pi* genes, while for each chromosome harboring the *Pi* genes (chromosome 2 for *Pib*, 6 for *Piz*, 11 for *Pik*, and 12 for *Pita*), 10 markers were selected. These 40 markers were located in regions of Nipponbare reference genome assembly IRGSP-1.0 where homologs of the *Pi* genes or *Pi* markers are localized (Supplementary Table S3). For *Pib*, the homolog sequence in Nipponbare (Os02g0818500) is localized on chromosome 2 from 35,113,637 bp to 35,116,084 bp. The *Piz* marker Z56592 is located on Nipponbare chromosome 6 at 11,677,565–11,677,843 bp and Os06g028700, which is indicated to be similar to the NBS-LRR type-R protein Nbs4-Pi, is localized from 10,387,793 bp to 10,390,465 bp. Considering that Nbs4-Piz-t is defined as Piz-t in the rice variety Toride [37], it cannot be excluded that Nbs4-Pi in Nipponbare is the homolog of *Piz* in Jefferson and *Piz-t* in Toride. The homolog of *Pik* in Nipponbare (Os11g0689100) is localized on chromosome 11 from 27,981,132 bp to 27,991,195 bp, while the *Pita* homolog in Nipponbare (Os12g0281300) is located on chromosome 12 from 10,606,359 bp to 10,611,917 bp. Considering the positions of the selected *Pi* genes (Supplementary Table S3), the selected additional markers were located in regions overlapping or adjoining to the target genes in order to improve the recovery of the RP genome in the genomic regions adjacent to the four *Pi* genes.

Seven lines, out of eight carrying four *Pi* genes, set enough seeds and were submitted to background selection. The background selection was also applied to 39 BC_3_F_2_ lines with four different combinations of three genes: *Piz + Pik + Pita, Piz + Pib + Pik, Piz + Pib + Pita,* and *Pib + Pik + Pita*. Twenty-three KASP markers out of 96 resulted in being polymorphic between DP and RP (Supplementary Table S3). With the exclusion of the *Piz* region on chromosome 6, all the chromosomes had at least one polymorphic molecular marker to assist the RP genome selection. Moreover, in the three regions corresponding to *Pib, Pik*, and *Pita*, respectively, on chromosomes 2, 11, and 12, there was at least one polymorphic marker (Supplementary Table S3).

KASP marker genotyping data were used to calculate the recovery percentage of the Vialone Nano genome in the BC_3_F_2_ lines subjected to background selection, using the formula:

(1 − (*number of heterozygous markers*/*total number of markers*)) × 100.

The recovery percentage values ranged from 65.22% to 95.65% (Supplementary Table S4) among lines. Interestingly, out of the seven BC_3_F_2_ lines with four *Pi* genes, four showed a recovery percentage of higher than 90% and one (06BC304F2127) higher than 95%. Recovery percentage values higher than 95% were also calculated for several lines carrying three pyramided genes.

### 2.5. Phenotyping

A phenotypic screening for blast resistance was performed using four multivirulent blast isolates to inoculate the original varieties carrying the four *Pi* genes: the DP SJKK, the BC lines carrying three or four *Pi* genes, together with Maratelli and Vialone Nano (RP) as susceptible controls (Table 4).

The two lines (154/05/3/154/A and 154/06/04/127) with four genes showed resistance against all four fungal strains. All the lines with three genes were also resistant to all the tested fungal strains, with some exceptions highlighted in three lines for which a few lesions were detected in one replicate.

Preliminary evaluations were carried out in a paddy field in 2022 for the line 154/06/04/127, which is under registration for commercial exploitation. Data related to amylose, biometrics of the kernels, and sowing to flowering time were in agreement with those obtained for the RP Vialone Nano, indicating that these traits were fully recovered in the introgression line (Table 5). The relevant difference recorded for yield was ascribable to a heavy incidence of the blast disease on the RP.

### 2.6. Checking for the Presence of the Pita2/Ptr Gene in the Introgression Lines

Katy was the original donor of *Pita* and also carries the tightly linked blast-R gene *Pita2/Ptr*, located on the pericentromeric region of rice chromosome 12 [38]. On the Nipponbare reference sequence, the *Pita* locus LOC_Os12g18360 (Os12g0281300) is located on chromosome 12 from 10,606,359 bp to 10,612,068 bp, which is indicated as *Pita* on RAP-DB (http://rapdb.dna.affrc.go.jp/download/irgsp1.html (accessed on 1 February 2016)). Conversely, LOC_Os12g18729 (Os12g0285100), corresponding to *Pita2/Ptr*, encompasses a chromosome 12 region from 10,822,534 to 10,833,768 bp [38,39]. Therefore, these two *Pi* genes are separated by only approximately 2 Mbp in a pericentromeric site where, most likely, meiotic recombination is reduced, raising the possibility that these two genes could have been co-transferred from Katy to SJKK. To verify this, a gene-specific KASP marker was designed for *Pita2/Ptr* (Table 2) and was used to genotype the alleles on SJKK, Katy, Vialone Nano, and the seven lines phenotypically evaluated for blast response. Results indicated that the five individuals carrying *Pita* have the same allele of Katy and SJKK at this marker, supporting that *Pita2/Ptr* was also pyramided in these lines.

## 3. Discussion

This work was focused on obtaining rice lines with pyramided *R* genes against *P. oryzae* in the background of the renowned Italian cv Vialone Nano by the application of MABC and KASP markers (the whole procedure is summarized in Figure S2). MABC represents a suitable procedure for the introgression of *R* genes in susceptible genomes, reducing the donor genome content into the elite variety [40]. Compared to traditional approaches of gene pyramiding, MABC has several advantages, including saving time, since a traditional approach would imply the availability and selection of the resistant genes using differential pathogen strains. It also minimizes the negative effects of linkage drag, since the background selection leads to the identification of lines with higher recovery of the RP genome [9,20].

In this work a pre-selection with a few foreground markers, followed by a final background selection on selected lines, was conducted. This strategy was demonstrated as more efficient than genome-wide background selection with high-throughput markers alone [41]. The high-throughput markers used in this work were assayed by KASP marker analysis based on SNPs, and are characterized by several advantages: high abundance of genomes, low cost, co-dominant inheritance, locus specificity, potential for high-throughput analysis, and relatively low genotyping error rates [42,43,44]. The KASP markers developed here can be complementary or alternative to other *Pi* markers (e.g., [7,16,20]) and can provide additional breeding tools for pyramiding effective blast-*R* genes in rice.

A low level of polymorphism was highlighted during the screening of the 96 KASP markers in the background selection: only 23 markers, over 96 (24%), were polymorphic between SJKK and Vialone Nano. This result can most likely be ascribed to the relatedness of the two varieties, as recent studies indicate that *japonica* accessions are highly related to each other, resulting in a slow linkage disequilibrium (LD) decay because of few historical recombination events [45,46]. LD decay, indeed, has been reported to range from 500 kb up to 2 Mb, in a temperate *japonica* background, and to 75 kb, in an *indica* background [46,47,48,49,50]. Even with a moderate coverage of markers, due to the low level of polymorphism, the application of the MABC scheme coupled with background selection allowed the identification of an elite line harboring four *Pi* genes with a 95.24% recovery percentage in just four years. In addition, several lines with three pyramided genes were identified as having RP values higher than 95%. Of these, two lines carrying two different combinations of three *Pi* genes (*Piz* + *Pib* + *Pita* and *Pib* + *Pik* + *Pita*) reached the recovery percentages of 95.45% and 91.30%, respectively, and a reduced number of heterozygous alleles.

The use of resistant cvs with multiple *R* genes is one of the most efficient and environmentally and economically sustainable methods of limiting disease incidence, leading to long-lasting resistance, and of defeating pesticide hazards [20,51]. The pyramiding of blast-*R* genes represents a useful tool to prevent infection and was applied to different rice cvs for up to four blast *R* genes/QTLs [20]. In fact, the probability that a pathogen may overcome an entire set of *R* genes is based on the possibility that the pathogen avirulence genes mutate and escape recognition by the corresponding *R* genes [52]. It is expected that, for asexual pathogens, accumulating several virulences will be a long process and that, due to fitness cost, multivirulent strains will not emerge due to lower competitiveness. The ascertainment of the effect of pyramiding on resistance durability would require the long-term monitoring of field resistance for the pyramided lines. However, reports of increased resistance (i.e., fewer lesions) provided by stacking different blast-*R* genes are available: rice pyramiding of *Pi1* + *Piz-5* + *Pita2* and *Pi-1* + *Pi-9* + *Pi-kh* enhanced blast resistance in comparison to a single gene [53,54,55]; nine *Pi* genes were introgressed individually or in combination of two by MABC, and the obtained lines demonstrated that *Pigm* and *Pid3* significantly enhanced resistance during the growth period [56]; Wu et al. [57] obtained pyramided lines carrying different alleles of the *Piz* locus (*Pigm*, *Pi40*, *Pi9*, *Pi2*, and *Piz*) combined with *Pi1*, *Pi33*, and *Pi54*, respectively, and demonstrated that polygene pyramided lines displayed more seedling and panicle blast resistance than monogenic lines.

The lines obtained in this work showed resistance to all four blast strains utilized for phenotypic evaluations and were chosen for their broad and complementary virulence pattern. No substantial differences were observed for the level of resistance of the lines with four genes in comparison to those with three genes, with the exclusion of three lines for which some blast symptoms were observed in one replicate. The results obtained for the lines with four genes (154/05/3/154/A and 154/06/04/127) agree with the levels of resistance observed for the lines containing the pyramided genes: BL1 (*Pib*) can confer resistance to NG0190, K1 (*Pita*) can confer resistance to TG0015, Kanto51 (*Pik*) can confer resistance to BN0013, while Zenith (*Piz*) can confer resistance to BN0040. Therefore, the presence of the four genes is expected to protect from the virulence functions of all four strains. For two lines showing pyramiding of *Piz*, *Pib*, and *Pik* (154/05/01/219/C and 154/05/11/65), resistance to isolating TG0015 was unexpected, given the absence of *Pita* (and *Pita2/Ptr*). This result deserves additional investigations to address the origins of this resistance. Epistasis or synergic effects might explain this behavior. For the other three lines, unexpected resistance responses were observed: in 154/05/01/185 the absence of *Pik* should confer no resistance to BN0013; similarly, in 154/05/11/135, the absence of *Pib* should result in susceptibility to NG0190; in 154/16/70/310, the absence of *Piz* did not explain the resistance to BN0040. Even though additional investigations are required to address these observations, the presence, in all these lines, of the broad-spectrum *Pita2/Ptr* gene could explain the extended pattern of resistance.

The original donors (Saber, Katy, Kusabue, and Jefferson) are resistant to all four blast strains used in this study, suggesting the presence of additional *Pi* genes in these genetic backgrounds, with respect to those for which they acted as donors. As an example, Jefferson has three *Pi* genes: *Pi-d(t)* and *Pik^h^-(t)*, tightly linked on chromosome 11; and *Piz*, located on chromosome 6 close to the centromere [58]. Furthermore, as mentioned before, Katy carries at least two *Pi* genes, *Pita* and *Pita2*/*Ptr*, tightly linked on the pericentromeric region of chromosome 12, and acting as a donor of both to SJKK and, consequently, to the lines tested for resistance.

In two lines (154/05/3/154/A and 154/06/04/127), four *R* genes were stacked; these data were then updated to five genes, after verification that *Pita2*/*Ptr* was also introgressed. This last gene provides a broad-spectrum resistance and represents a non-NLR gene encoding a novel R protein containing an Armadillo repeat domain [38,39]. The verification of whether this level of pyramiding will provide durable resistance will require the long-term monitoring of the resistance under blast pressure in field conditions.

Vialone Nano is a traditional and renowned Italian variety, grown on several thousands of hectares and also having a Protected Geographical Indication (in Italy, denominated IGP). Nonetheless, it is one of the most blast-susceptible Italian cvs [8,59]. Although treated with fungicides during the growing season, Vialone Nano often suffers a serious yield penalty when exposed to moderate pathogen pressure, making the sustainability of this cultivation very uncertain. The introduction of multiple *Pi* genes effective against field-blast population [7,60] will most likely allow relief from or solution of the problems related to blast incidence in this rice variety.

## 4. Materials and Methods

### 4.1. Plant Material and DNA Extraction

Rice genotypes Katy, Saber, and Jefferson, original donors of *Pita, Pib*, and *Piz*, respectively, were obtained from the USDA National Small Grains Collection, Aberdeen, WA, USA; Kusabue, harboring *Pik*, was obtained from the National Institute of Agrobiological Sciences (NIAS), Japan. The elite Italian variety Vialone Nano (RP) was provided by CREA-CI, Vercelli, Italy; DP SJKK, harboring the four *Pi* genes [17], was derived from Bertone sementi SpA, Terruggia, Italy. It was obtained from a pyramiding program, assisted by molecular markers, using Katy, Saber, Jefferson, and Kusabue as *Pi* donors [17].

Seeds were surface-sterilized by washing in 70% ethanol for 1 min, followed by another washing with 30% bleach for 30 min and three rinses with distilled water for 5 min. Seeds were then germinated in Petri dishes on wet paper. After one week, seedlings were planted in pots filled with peat moss (225 L), vermiculite (125 L), calcium carbonate (225 g), and osmocote exact 3–4 M (700 g) and watered.

DNA was extracted from leaves of two-week-old plants from each *Pi* donor, DP, RP, the F_1_, BC_n_F_1_, and self-pollination (BC_3_F_2_) progenies (see below), according to the method described by [61].

### 4.2. Molecular Marker Development and Analysis

Some of the markers utilized in the present work to assess the presence of resistant alleles at the *Pib* and *Piz* loci were already available (see below), while for *Pita* and *Pik*, markers were derived from Nipponbare homologous loci. For all the markers, the general strategy was based on the amplification of genomic fragments from both the donor SJKK (as well as from the original donors: Saber for *Pib*, Jefferson for *Piz*, Katy for *Pita*, and Kusabue for *Pik*) and the RP Vialone Nano, followed by sequencing and searching for sequence polymorphisms to develop co-dominant diagnostic molecular markers.

The sequence of *Pita* [35] was obtained by searching for the locus ID LOC_Os12g18360 (Os12g0281300, chr12: from 10.606.359 bp to 10.612.068 bp) on the Gramene database (http://www.gramene.org/, (accessed on 1 February 2016)). Overlapping primers (Supplementary Table S1) were designed by Vector NTI Software (Thermo Fisher Scientific), to achieve ~500-bp-long overlapping amplicons.

The sequences corresponding to the *Pik-1* and *Pik-2* alleles in Nipponbare were indicated as *Pik5-NP* and *Pik6-NP*, respectively, by [34]. Both genes are needed to confer the *Pik* resistance [28]. The sequence of the *Pik-1* locus in Nipponbare, LOC_Os11g46200.1 (Os11g0688832, chr11: 27,981,132–27,991,195 bp), corresponding to the *Pik5-NP* gene indicated by [34], was obtained from the Gramene database (http://www.gramene.org/ (accessed on 1 February 2016)). Overlapping primers (listed in Supplementary Table S1) were designed, as described above, to amplify *Pik1* sequences in the donor(s) and receiver(s). A marker was first obtained for *Pik1* using primers Pik1-10f/r designed from LOC_Os11g46200.1 (Os11g0688832), but it was not suitable for the KASP marker assay (detailed in the Results Section 2.1). To develop useful KASP markers, the *Pik-2* sequence (available in GenBank—accession number HM048900; [34]) was used to search the Vialone Nano *Pik2* homolog sequences by a BlastN search within a custom Vialone Nano genome assembly (unpublished data). The two sequences were aligned using Sequencing Analysis Software 6 (Applied Biosystems Inc., Foster City, CA, USA), and regions carrying putative polymorphisms were targeted by designing additional primers, by means of Primer3Plus (http://www.bioinformatics.nl/cgi-bin/primer3plus/primer3plus.cgi (accessed on 15 November 2018)), which were used to amplify overlapping regions of *Pik2* in SJKK and Vialone Nano (Supplementary Table S1). Primers Pik2-2AEf/r (listed in Table 1), designed to amplify a region from 3869 bp to 4322 bp of HM048900, allowed the identification of SNP-based markers.

Primers for *Pib* and *Piz* were already available in the literature [7,33,37]. Figure 2 shows the localization of the *Pi* markers with respect to the positions of *Pita, Pik*, and *Pib* homolog genes in Nipponbare. *Piz* markers (Z6050 and Z56592, obtained from [33,37], respectively), were localized at 9,916,006–9,916,133 bp and 10,407,985–10,408,277 bp, respectively, in Nipponbare. The position of *Piz* in Nipponbare was inferred by blasting the *Piz* alleles *Pi-zt*, *Pi2,* and *Pi9* on the Os-Nipponbare-Reference-IRGSP-1.0 genome assembly (http://rapdb.dna.affrc.go.jp/download/irgsp1.html (accessed on 15 June 2017)), allowing the identification of LOC_Os06g17900 (Os06g0286700, 10,387,973–10,390,465 bp) as the putative Nipponbare allele for *Piz*, *Pizt*, *Pi2*, and *Pi9*. The position of *Piz* markers Z6050 and Z56592 with respect to the *Piz* allele in Nipponbare is indicated in Figure 2.

Primers to amplify the *Pita2*/*Ptr* locus were designed using the Primer3Plus program on the *Ptr* sequence cloned in Katy (Genebank ID: MG385185.1). The analysis was focused on less-conserved regions (UTRs and introns), in order to increase the probability of detecting polymorphisms between the different varieties.

For the amplification of entire gene fragments (for *Piz* and *Pita*), PCR reactions were performed in a total volume of 10 μL containing 20 ng of genomic DNA, 500 nM forward and reverse primers, 1.5 mM MgCl, 5% DMSO, 0.25 mM dNTPs, and 1U GoTaq DNA Polymerase (Promega, Madison, WI, USA). A touchdown program was applied as follows: denaturation at 94 °C for 3 m, 10 cycles at 94 °C for 45 s, from 60 °C to 55 °C for 45 s, reducing the annealing temperature of 0.5 °C for each cycle, and 72 °C per 45 s, 24 cycles at 94 °C for 45 s, 51 °C for 45 s, and 72 °C for 45 s, with a final extension at 72 °C for 10 m.

Some markers were utilized for both MABC and KASP (Table 1). For the initial polymorphism searches, carried out by comparing the RP with the donors, PCRs were performed in a 20 µL reaction mix, containing 1.5 mM MgCl_2_, 0.25 mM of each dNTP, 0.5 µM of each primer, 5% DMSO, 1U GoTaq^®^ G2 Flexi DNA polymerase (Promega, Madison, WI, USA), and 40ng of genomic DNA. The PCR program comprised 1 cycle of 4 m at 94 °C, 35 cycles of 30 s at 94 °C, 40 s at the annealing temperature indicated in Table 1, 1 m at 72 °C, and a final extension 72 °C for 7 m.

All the PCR products were purified after electrophoresis on 1% agarose gel by a Wizard^®^ SV Gel and PCR Clean-Up System (Promega, Madison, WI, USA) and directly sequenced using Big dye terminator v1.0 in each direction, using the primers indicated in Table 1 and in Supplementary Table S1. A capillary electrophoresis instrument (3130XL Genetic Analyzer; Applied Biosystems Inc., Foster City, CA, USA) was utilized. SNPs were identified by aligning the RP and RP sequences using Sequencing Analysis Software 6 (Applied Biosystems Inc., Foster City, CA, USA).

Restriction enzymes suitable for SNP recognition were identified using the ‘‘Restriction Enzyme Site Mapper version 3” software (http://www.restrictionmapper.org/ (accessed on 20 February 2016)). PCRs for marker scoring on cross or BC progenies were performed as for the polymorphism screen. For the CAPS and dCAPS marker scorings, 10 µL of unpurified PCR products were digested overnight in a 15 µL volume containing 1× restriction enzyme buffer and 2.5U restriction enzyme, following the manufacturer’s instructions. The resulting fragments were visualized on 2% agarose gels.

### 4.3. Foreground Selection by KASP Marker Assays

Two thousand and five hundred BC_3_F_2_ seeds, together with those of the parents SJKK and Vialone Nano, were sown as described in Section 4.1. DNA extracted from each plant was screened in foreground selection by KASP (Kompetitive Allele-Specific) markers. The sequences listed in Table 2 and shown in Supplementary Figure S1 were used to develop KASP marker assays corresponding to the SNP present in each sequence of *Piz, Pib, Pik*, *Pita*, and *Pita/Ptr* genes, indicated in red in Table 2 and Supplementary Figure S1. The selected SNPs were sent to LGC Genomics UK (https://www.lgcgroup.com/ (accessed on 1 February 2023)) for primer design. For these SNPs, 250 bp flanking the candidate SNPs on either side were provided to LGC. The foreground analyses for *Piz, Pib, Pik*, and *Pita* genes were carried out by the LGC group. A subsequent foreground analysis for evaluating the presence of the *Pita2/Ptr* gene linked to the *Pita* gene on chromosome 12 was carried out on the selected introgression lines 159/16/70/310, 154/06/04/127, 154/06/1/185, 154/05/3/154A, 154/05/219/C, 154/05/11/135, 154/05/11/65, the DPs Katy and SJKK, and the RP Vialone Nano. The allelic discrimination assay for the *Pita2/Ptr* gene was tested in a 96-well format and established as 10 μL reactions (4.86 μL of template DNA (50 ng), 5.0 μL of 2× Low Rox Kasp mix, and 0.14 μL of primer mix). PCR was performed on a BIORAD-CFX OPUS device in accordance to the following protocol: pre-read stage at 60 °C for 60 s, hot start at 94 °C for 15 min, 10 touchdown cycles (94 °C for 20 s; touchdown at 61 °C, −0.6 °C per cycle, 60 s) and then 26 cycles of amplification (94 °C for 20 s; 55 °C for 60 s), 3 cycles of amplification (94 °C for 20 s; 57 °C for 60 s), and a post-read stage at 40 °C for 60 s.

### 4.4. Background Selection by KASP Marker Assays

The background selection was carried out by LGC (https://www.lgcgroup.com/ (accessed on 1 February 2023)) on 46 BC_3_F_2_ individuals selected on the basis of the foreground selection results, and on DP and RP, using 96 KASP markers evenly spaced over the 12 rice chromosomes. On average, one SNP every 1–5 Mb (depending on chromosome size and markers available) was selected from the commercially available KASP markers, resulting in an average of seven SNPs per chromosome. For chromosomes 2, 6, 11, and 12, bearing the introgressed *Pi* genes, 10 KASP markers were selected in order to include about 3 additional markers (spaced at a distance lower than 1 Mb) in the genomic regions where *Pi* genes are localized. The 96 markers employed in the analysis are listed in Supplementary Table S3.

### 4.5. Phenotyping

A resistance screening was performed, as described by [62] on seven BC_3_F_4_ lines selected on the bases of the recovery of the RP genome, using four multivirulent blast isolates (BN0013, BN0040, NG0190, and TG0015), each being avirulent to one of the four *Pi* introgressed genes (Table 4). The four initial DPs (Jefferson, Katy, Kusabue, and Saber), differential varieties carrying *Pi* genes (BL1 for *Pib*, K1 for *Pita*, Kanto51 for *Pik*, and Zenith for *Piz* [63]), and the susceptible cvs Maratelli and Vialone Nano were used as controls and were evaluated together with the lines carrying four and three *Pi* genes. The fungal isolates were grown on rice flour agar medium (15 g agar, 20 g rice flour, 2.5 g yeast extract, 1 L of water) for 7 days at 27 °C under fluorescent light (12 h photoperiod). All cvs and lines were sown in a tray (30 × 45 cm) filled with peat soil. Plants were grown in a greenhouse for 4–5 weeks (4-leaf stage) and inoculated by spraying spore suspensions (20 mL of suspension at 20,000 spores/mL per tray). Due to the limited number of seeds per each pyramided line, inoculation was performed a second time on the same plants after removing diseased leaves. Resistance was assessed at 7 days post-inoculation using a 1–6-scale rating system based on lesion type as described by [62]. Plants with scores 1 to 3 (absence of lesions or non-sporulating lesions) were considered as resistant (R), whereas interactions with scores 4 to 6 (sporulating lesions) were considered as susceptible (S). The preliminary evaluation in a paddy field for yield and quality traits of line 154/06/04/127 in comparison with the RP Vialone Nano was carried out in 2022 in Lucedio (Vercelli, Italy) in replicated plots of 50 m^2^ each. After harvest, analyses of amylose content, grain size, and thousand kernel weight were performed on the same materials using previously defined procedures [64].

## 5. Conclusions

Progress in rice research has paved the way for overcoming biotic stresses in order to retain high yields, improving the performance of highly appreciated rice cvs, such as Vialone Nano, an IGP Registered Trademark in Northern Italy, but whose cultivation may be hampered by its high susceptibility to blast. Most *Pi* donor parents for European rice varieties have up to now been tropical varieties that do not fit with European climate conditions [6,7,8,17]. Thanks to the MABC scheme integrated by the molecular KASP markers assays, a temperate *japonica* variety with a broad spectrum of resistance to blast was obtained in the Vialone Nano genomic background in the relatively short period of four years, retaining the typical features of the RP in terms of amylose content and kernel biometric traits, and showing, in a preliminary field test with heavy blast occurrence, increased yield compared to the RP. In addition, this line might be used as a donor in future breeding programs to yield other European elite varieties resistant to *P. oryzae*. Disease screening demonstrated the ability of this variety to withstand blast, as it has a complete and broad-range resistance, a very important trait for plant breeders and farmers to guarantee sustainable production.

## 6. Patents

The donor SJKK and all its derivatives are protected by a patent certificate for industrial invention (Italian Ministry of Economic Development N.102015000076118, 5 May 2018), owned by Bertone Sementi S.p.A.

## Figures and Tables

**Table 1:** List of the primers. For each primer, the name, sequence, annealing temperature (Ta), polymorphism type, restriction enzyme (RE, in the case of CAPS and dCAPS markers), gene, reference, and application are reported. InDel = Insertion/Deletion; SNP = Single Nucleotide Polymorphism; CAPS = Cleaved Amplified Polymorphic Sequence; dCAPS = derived Cleaved Amplified Polymorphic Sequence; Pres/abs = presence/absence.

Primer Name	Sequence	Ta (°C)	Polymorphism Type	RE	Gene	Reference	Application in:	
							MABC	KASP Marker /Sequencing
Pib5 f Pib5 r	CCTACTGCTCTCGCTCCGAATTCC CAGAATTTTGTCAGGAACCTGCC	58	InDel and SNPs	-	*Pib*	[7]		x
Pib3 f Pib3 r	AGTAGTATCTCCCTACTCACACGACAC GGATGACCTGAACTGAAACTCACAGT	58	dCAPS	DdeI	*Pib*	[7]	x	
Z6050 f Z6050 r	CCCGAGCACTTGCAGATCTTGGGCGAAGCA ATCTCTCCGGCACGACCGAAGC	60	dCAPS	SphI/PaeI	*Piz*	[7,33]	x	
Z56592 f Z56592 r	GGACCCGCGTTTTCCACGTGTAA AGGAATCTATTGCTAAGCATGAC	60	SNPs	-	*Piz*	[36]		x
Pik2-2AE f Pik2-2AE r	TCCTTAGCCCTGTCAACTGA TGCTGGATCTTGAGGACTGG	53	SNPs	-	*Pik2*	This work		x
Pik1-10 f Pik1-10 r	ACTGTAGTGCATACCATTGG GAGTGCTCCCCACATACAA	50	Pres/abs	-	*Pik1*	This work	x	
Pita-10 f Pita-10 r	TATCTTGCAAATGCGTCCG GCCAAGAAGATGATCAGCA	60	CAPS	HincII	*Pita*	This work	x	x
Pita2/Ptr -5 f Pita2/Ptr-5 r	TTGCTTGTTCGGATCAGTGC TGTTGCTGATCGTCTTGCTG	57	SNPs		*Pita2/Ptr*	This work		x

**Table 2:** Sequences for each *Pi* gene marker used for the KASP marker assay development. The selected SNPs are indicated in red. Within the sequences, the IUPAC Code characters R, W, S, Y, M are used, indicating the presence of unselected other SNPs following the encoding R = A/G, W = A/T, S = C/G, Y = C/T, M = A/C. The N character indicates presence of INDEL.

Amplicon	Donor Parent Sequence SJKK	Recurrent Parent Sequence Vialone Nano
*Pib5*	TCAAGAGAATTTGAGAAGCAAGAGAGGC TCTGATACCAGATTGTCAGGATCTCAAGAA ATCARCAANGARMAACAAGAACACACAA GGATTCAGGCAACTAGTTTGGATTGATCTG CTCCAACCCAACAGG	TCAAGAGAATTTGAGAAGCAAGAGAGGC TCTGATACCAGATTGTCAGGATCTCAAGAA ATTARCAANGARMAACAAGAACACACAA GGATTCAGGCAACTAGTTTGGATTGATCTG CTCCAACCCAACAGG
*Z56592*	CGATGTTCGAGAGCCCATGGATGTTTAGTT GTTTAGACATGGTGTTGGACGGTCGAATGG TGGGCCTGTTGTAGGTATGGTGGCATCTGG CAACCAGTCAT	CGATGTTCGAGAGCCCATGGATGTTTAGTT GTTTAGACATGGTGTTGGACAGTCGAATGG TGGGCCTGTTGTAGGTATGGTGGCATCTGG CAACCAGTCAT
*Pik2-2AE*	CCTTGAGGYGACGGGTTTTTMATTGSCTTMTT WCTTTTTTCTTGAGGCAACCTCAGCCCCTTCC GTGTGTTTTTRTTCCCYCCAAGTATGCTAMTG ATCCGTTTTAGCTGGMCTAYWGACTTAGGCA GSTCCYYGACATATGTCTCCCTTATGT	CCTTGAGGYGACGGGTTTTTMATTGSCTTMTT WCTTTTTTCTTGAGGCAACCTCAGCCCCTTCC GTGTATTTTTRTTCCCYCCAAGTATGCTAMTG ATCCGTTTTAGCTGGMCTAYWGACTTAGGCA GSTCCYYGACATATGTCTCCCTTATGT
*Pita-10*	GTCAGCGACAGAAACCGGCGGCGTTCGTTG CCGGCGGAGTCCTCGCGATCGTCGTCGTCGT CTTCTTCTCTCGGCCTCGAGCTCGAGGTGCG CCTGCCAAGATGGTAGCTC	GTCAGCGACAGAAACCGGCGGCGTTCGTTG CCGGCGGAGTCCTCGCGATCGTCGTCGTCGA CTTCTTCTCTCGGCCTCGAGCTCGAGGTGCG CCTGCCAAGATGGTAGCTC
*Pita2/Ptr*	TATACACAGTAGACAATATTGGATTGAGTTT CTGATGAACACAGTAGGAATTCTTCTCAATA CGGTGTGTGCGTGTAGAGAATAATCAACAAT GGTACGTCGTGCTGCTTGCCTCGGCGCACGG CAACA	TATACACAGTAGACAATATTGGATTGAGTTT CTGATGAACACAGTAGGAATTCTTCTCGATA CGGTGTGTGCGTGTAGAGAATAATCAACAAT GGTACGTCGTGCTGCTTGCCTCGGCGCACGG CAACA

**Table 3:** Number of plants for each *Pi* gene combination out of 2364 *BC*_3_*F*_2_ screened lines.

Locus				Number of Plants
*Piz*	*Pib*	*Pik*	*Pita*	
+	+	+	+	8
+	+	+	-	5
+	+	-	+	27
-	+	+	+	40
+	-	+	+	10
-	+	-	+	147
+	-	-	+	45
-	+	+	-	44
-	-	+	+	65
+	-	+	-	51
+	+	-	-	19
-	-	-	+	241
-	+	-	-	139
+	-	-	-	176
-	-	+	-	283

**Table 4:** Screening of resistance to 4 multivirulent strains of *P. oryzae*. The original donors of the *Pi* genes are in bold. ND = not determined, because most of the plants were resistant in most of the experiments but a few lesions were observed in at least one replicate.

Variety	*Pi* Gene(s)	Phenotypic Scores			
		BN0013	BN0040	NG0190	TG0015
**Saber**	*Pib*	R	R	R	R
BL1	*Pib*	S	S	R	S
**Katy**	*Pita* and *Pita2/Ptr*	R	R	R	R
K1	*Pita*	S	S	S	R
**Kusabue**	*Pik*	R	R	R	R
Kanto 51	*Pik*	R	S	S	S
**Jefferson**	*Piz*	R	R	R	R
Zenith	*Piz*	S	R	S	S
Vialone Nano	-	S	S	S	S
Maratelli	-	S	S	S	S
154/05/3/154/A	*Piz + Pib + Pik + Pita + Pita2*	R	R	R	R
154/06/04/127	*Piz + Pib + Pik + Pita+ Pita2*	R	R	R	R
154/05/01/219/C	*Piz + Pib + Pik*	R	R	R	R
154/05/01/185	*Piz + Pib + Pita+ Pita2*	R	R	R	ND
154/05/11/135	*Piz + Pik + Pita+ Pita2*	R	R	R	R
154/05/11/65	*Piz + Pib + Pik*	ND	R	R	ND
154/16/70/310	*Pib + Pik + Pita+ Pita2*	R	R	R	ND

**Table 5:** Comparison of some agronomic, biometric, and qualitative traits for a selected introgression line (154/06/04/127) and the RP Vialone Nano.

Parameters	Line 154/06/04/127	Vialone Nano
Amylose (% d.m.)	25.90	26.80
Grain length, L—milled (mm)	5.57	5.51
Grain width, W—milled (mm)	3.13	3.21
L/W (milled)	1.78	1.72
Grain length, L—hulled (mm)	6.09	6.12
Grain width, W—hulled (mm)	3.30	3.56
L/W (hulled)	1.85	1.72
Thousand kernel weight (g)	37	36
Sowing–flowering (days)	102	103
Grain yield (t/ha)	9.1	5.8

## Data Availability

The data presented in this study are available on request from the corresponding author.

## Supplementary Materials

The following Supplementary files are available online at https://www.mdpi.com/article/10.3390/plants12040757/s1, Table S1: List of primers used to amplify overlapping sequences of the *Pita*, *Pik*, and *Pita2/Ptr* alleles of the Vialone Nano recurrent parent. Table S2: Results of the foreground selection of the BC_3_F_2_ lines carried out with the KASP markers. Table S3: Chromosomal and physical position of the KASP markers selected for the background selection. Table S4: Alleles of the KASP markers used for the background selection and percentage of RP observed in the Vialone Nano introgression lines. Figure S1: SNPs polymorphisms between the *Pi* genes donor SJKK and Vialone Nano (VN) for *Pita*, *Piz*, *Pib*, and *Pik* blast-resistance gene markers. Figure S2: Molecular markers used along the steps of MABC selection.

## Abbreviation

MABC: Marker-Assisted BackCrossing; BC: BackCross; RP: recurrent parent; DP: donor parent; InDel: Insertion/Deletion; SNP: Single Nucleotide Polymorphism; CAPS: Cleaved Amplified Polymorphic Sequence; dCAPS: derived Cleaved Amplified Polymorphic Sequence; Pres/abs: presence/absence; Ta: Annealing Temperature; RE: restriction enzyme; KASP: Kompetitive Allele Specific; QTLs: quantitative trait loci; cv: cultivar.
