# Supplementary figures and images for: Marker-Assisted Pyramiding of Blast-Resistance Genes in a japonica Elite Rice Cultivar through Forward and Background Selection
Source: Plants (Basel). 2023 Feb 8;12(4):757. doi: 10.3390/plants12040757 (PMC9963729; doi:10.3390/plants12040757)

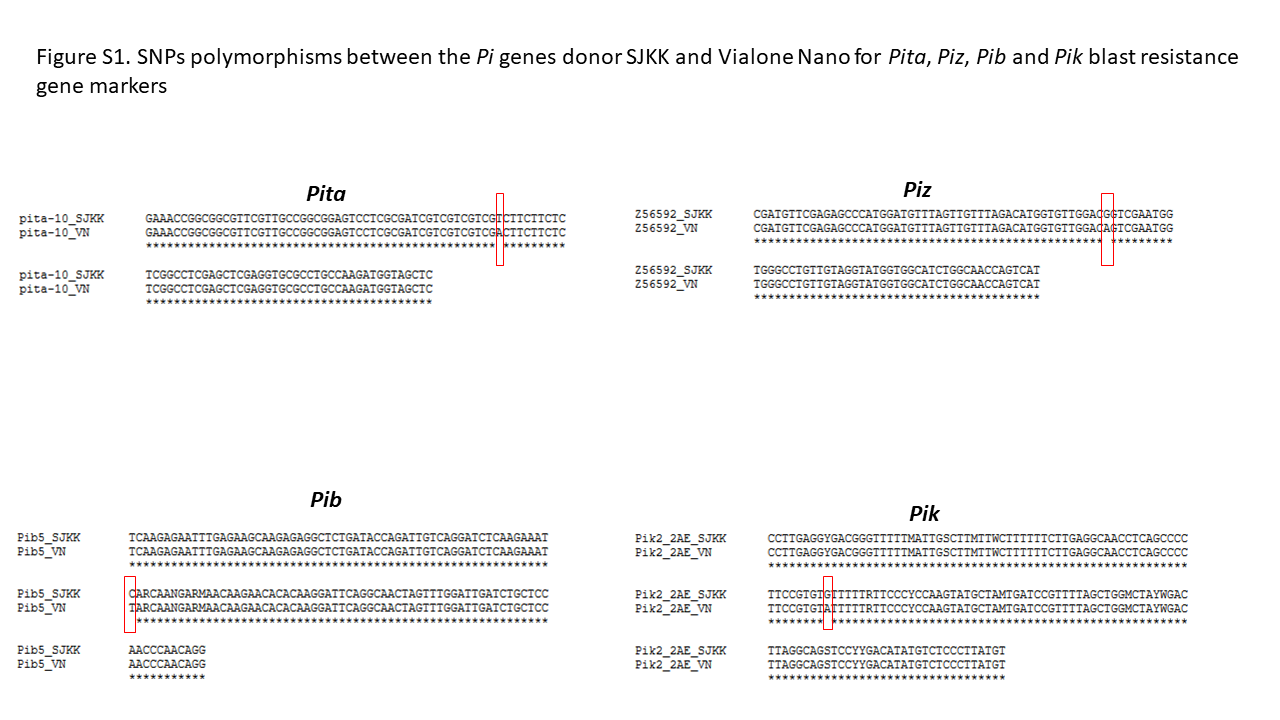

Supplement: Supplementary file 1 [file plants-12-00757-s001.zip › plants-2197078-supplementary/Supplementary Material Zampieri et al/Figure S1.tif]

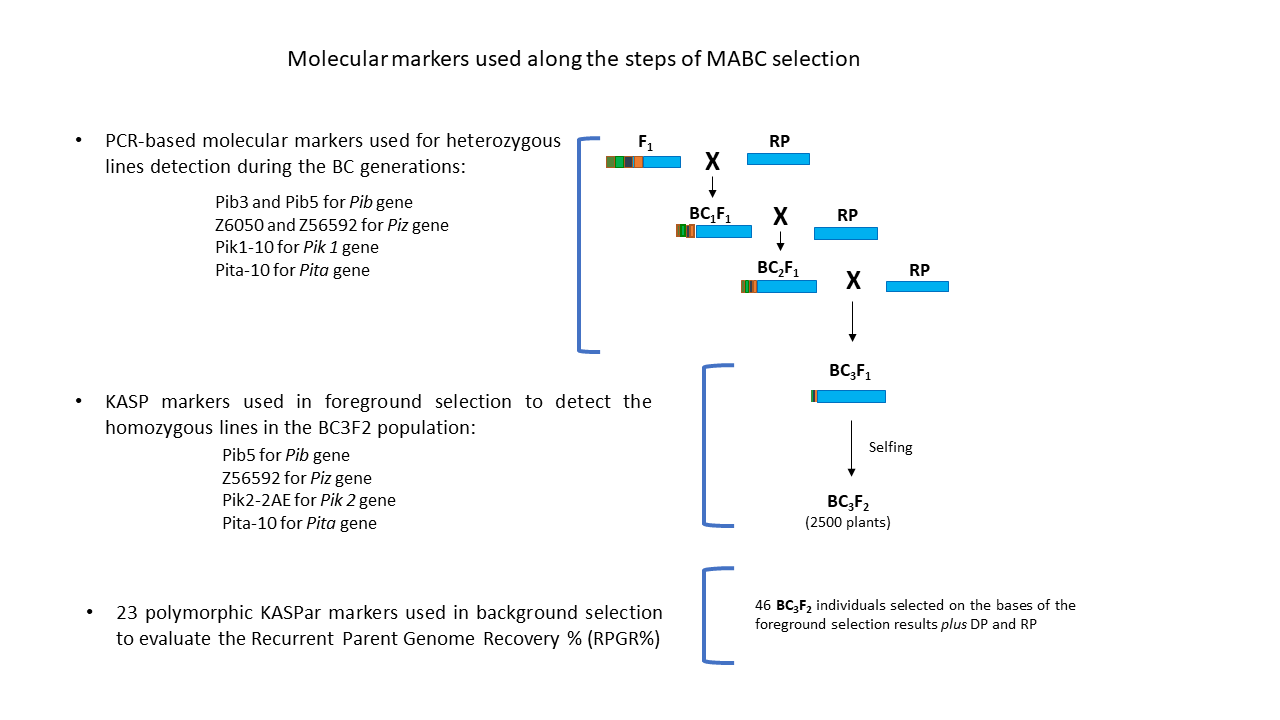

Supplement: Supplementary file 1 [file plants-12-00757-s001.zip › plants-2197078-supplementary/Supplementary Material Zampieri et al/Figure S2.tif]
